# The effects of NAD+ precursor (nicotinic acid and nicotinamide) supplementation on weight loss and related hormones: a systematic review and meta-regression analysis of randomized controlled trials

**DOI:** 10.3389/fnut.2023.1208734

**Published:** 2023-10-03

**Authors:** You Baichuan, Marcela Gomes Reis, Sogand Tavakoli, Navideh Khodadadi, Mohammad Hassan Sohouli, Nathalia Sernizon Guimarães

**Affiliations:** ^1^SDU-ANU Joint Science College, Shandong University, Weihai, China; ^2^Master in Health Science at Faculdade Ciências Médicas de Minas Gerais, Belo Horizonte, Minas Gerais, Brazil; ^3^OPENS: Observatory of Epidemiology, Nutrition and Health Research, Faculdade Ciências Médicas de Minas Gerais/FELUMA, Belo Horizonte, Minas Gerais, Brazil; ^4^Student Research Committee, Department of Clinical Nutrition and Dietetics, Faculty of Nutrition and Food Technology, Shahid Beheshti University of Medical Sciences, Tehran, Iran; ^5^Faculdade Ciências Médicas de Minas Gerais, Belo Horizonte, Minas Gerais, Brazil

**Keywords:** NAD+ precursor, niacin, obesity, obesity hormone, weight loss

## Abstract

**Background:**

Despite the fact that obesity and overweight are serious major health problems worldwide, fighting against them is also considered a challenging issue. Several interventional studies have evaluated the potential weight-reduction effect of nicotinamide adenine dinucleotide (NAD+) precursor. In order to obtain a better viewpoint from them, this study aimed to comprehensively investigate the effects of NAD+ precursor supplementation on weight loss, adiponectin, and leptin.

**Methods:**

Scopus, PubMed/Medline, Web of Science, Cochrane, and Embase databases were searched using standard keywords to identify all controlled trials investigating the weight loss and related hormones effects of NAD+ precursor. Pooled weighted mean difference and 95% confidence intervals were achieved by random-effects model analysis for the best estimation of outcomes.

**Results:**

Twenty two treatment arms with 5,144 participants’ were included in this systematic review and meta-regression analysis. The pooled findings showed that NAD+ precursor supplementation has an effect on lowering BMI (weighted mean difference (WMD): −0.19 kg/m2, 95% confidence interval (CI): −0.29 to −0.09, *p* < 0.001) and increasing adiponectin (WMD: 1.59 μg/mL, 95% CI: 0.49 to 2.68, *p* = 0.004) in humans compared with control groups. However, no significant effect was observed on body weight and leptin. There was a significant relationship between doses of intervention with changes in BMI. In addition, subgroup analysis showed that BMI reduction was greater when receiving nicotinic acid (NA) supplementation than nicotinamide (NE) supplementation.

**Conclusion:**

NAD+ precursor had significant effects on weight management with the reduction of BMI and increasing adiponectin.

## Introduction

Overweight and obesity are defined as abnormal or excessive fat accumulation that presents a risk to health. A body mass index (BMI) over 25 is considered overweight, and over 30 is obese. The issue has grown to epidemic proportions, with over 4 million people dying each year as a result of being overweight or obese in 2017 according to the global burden of disease (1). Adiposity, particularly abdominal adiposity, is associated with an increased risk of most chronic diseases such as type 2 diabetes (T2DM), cancer, cardiovascular disease (CVD), atherosclerosis, and metabolic syndrome (2). Moreover, low-grade chronic inflammation linked to adiposity, along with adipocyte development and a change in the pattern of activity of adipokines especially, leptin and adiponectin, leads to adipose tissue (AT) dysfunction (3). Following these changes, serious disorders are generally observed, such as a decrease in mitochondrial biogenesis and an increase in oxidative stress and inflammation (4–6). Therefore, one might hypothesise that elements such as nicotinamide adenine dinucleotide (NAD) + and their related components, which regulate mitochondrial function, metabolism, cellular stress response, carbohydrates and fatty acids synthesis, ATP generation, and ketogenesis, have been significantly involved in the adjustment of metabolic complications associated with obesity (7–9). NAD+ is synthesised in two major pathways in the human body: *de novo* synthesis and salvage, the latter of which has a more effective role in maintaining levels of NAD+ in the body. The compounds that are created from the salvage pathway are called NAD+ precursors (10). Furthermore, the presence of Sirtuins, NAD + -dependent protein deacetylases (SIRT1–7), is crucial for NAD + 's biological function in humans (11). Studies have shown that NAD+ precursors and Sirtuins gene expression were significantly down-regulated in obese subjects (12–14), whereas their expression increased progressively in subjects with a weight loss programme (15). However, the effects of NAD+ precursor supplementation on weight and other related factors, as well as adipokines, are still not completely clear. Therefore, the objective of this systematic review and meta-analysis of randomized controlled trials is to analyze and evaluate the effects of NAD+ precursor supplementation on weight loss and obesity hormone.

## Methods

### Search strategy

The Preferred Reporting Items for Systematic Review and Meta-analysis (PRISMA) criteria were followed for conducting this study (16). Without regard to language or time restrictions, a thorough search was carried out in the PubMed/MEDLINE, Web of Science, SCOPUS, and Embase databases from the beginning to February 2023. Additionally, similar papers and gray literature were considered in the search. Medical subject headings (MeSH) and Emtree (Embase subject headings) were selected to search the online databases, as follow: (“NAD” OR “NAD precursor” OR “Nicotinic Acids” OR “Niacin” OR “Niacinamide” OR “Nicotinamide Mononucleotide”) AND (“weight” OR “Waist Circumference” OR “Body Mass Index” OR “Adiponectin” OR “Leptin”) AND (“Clinical Trials as Topic” OR “Cross-Over Studies” OR “Double-Blind Method” OR “Single-Blind Method” OR “Random Allocation” OR “Clinical Trial”) (The specific search strategy is described in the Supplementary Appendix S1). The reference lists of the publications retrieved and linked review studies were manually searched to identify potentially overlooked qualifying trials.

### Eligibility criteria

Using titles, abstracts, or the complete texts of the research, two writers separately removed duplicate articles before finding and reviewing relevant publications. In the end, the papers were separated based on the following standards: (1) Randomized clinical trials studies; (2) NAD+ precursor supplementation (nicotinic acid (NA) or nicotinamide (NE) supplementation) was given as an intervention in individual’s aged 18 and over; and 3) baseline and post measurements in both group (intervention and control) weight, BMI, adiponectin, and leptin were recorded. The most recent or longest follow-up period was used when a research revealed results at more than one follow-up time. Studies with duplicated data, studies with ambiguous information, studies in which NAD+ precursor was used as an intervention alongside other commonly prescribed medications, non-randomized trial designs, animal studies, studies without a control group, reviews, and meta-analysis studies were also excluded. The PICOS criteria for inclusion and exclusion of studies were as follows. Population: individual’s aged 18 and over; Intervention: NAD+ precursor supplementation (nicotinic acid (NA) or nicotinamide (NE) supplementation); Comparator: other intervention or placebo; Outcomes: weight, BMI, adiponectin, and leptin; Study design: randomized clinical trials studies.

### Data extraction

The qualifying studies were examined by two authors independently. The first author’s name, the study’s location, the year it was published, the sample size (for the intervention and control groups), the participant characteristics (such as the percentage of men, the participant’s BMI, age, and health status), the type of outcomes, duration of the intervention, the dosage and type of the intervention, and the means and standard deviations (S.D.s) of the intended outcomes at baseline, post-intervention, and/or changes between baseline and post-intervention, were all extracted.

### Quality assessment

The details of the evaluation of the study’s quality are presented in Table 1. Using the Cochrane risk-of-bias test for randomized trials (RoB 2), version 2, the quality of the included RCTs was methodologically evaluated (17). Based on the following potential sources of bias: blinding of outcome assessment, allocation concealment, participant and staff blinding, random sequence generation, incomplete outcome data, selective reporting, and other bias, two authors independently rated each study as having a low, high, or unclear risk of bias. A “High risk” rating indicates significant bias that may invalidate the results. These studies have serious errors in design, analysis, or reporting; have large amounts of missing information; or have discrepancies in reporting. Studies are assessed as at unclear risk of bias when too few details are available to make a judgement of ‘high’ or ‘low’ risk; when the risk of bias is genuinely unknown despite sufficient information about the conduct; or when an entry is not relevant to a study (for example because the study did not address any of the outcomes in the group of outcomes to which the entry applies). A “Low risk” study has the least bias, and results are considered valid. Any discrepancies were discussed with a third author in order to come to a consensus. The GRADE (Grading of Recommendations Assessment, Development, and Evaluation) grading method was also used to evaluate the quality of the current analytic research (18). A reliable 10-point assessment system that assesses elements affecting study quality is the GRADE checklist. This scale has seven components: (1) risk of bias, (2) precision, (3) heterogeneity, (4) directness, (5) publishing bias, (6) funding bias, and (7) study design.

**Table 1:** Risk of bias assessment according to the Cochrane collaboration’s risk of bias assessment tool.

Study, Year (reference)	Random sequence generation	Allocation concealment	Blinding of participants and personnel	Blinding of outcome assessment	Incomplete outcome data	Selective reporting	Overall assessment of risk of bias
El-Kady et al. (2022)	Low	Low	Low	Low	Unclear	Low	Low
Liao et al. (2021)	Low	Unclear	Low	Low	Unclear	Low	Unclear
Canner et al. (2003)	Low	Low	Low	High	Unclear	Low	Unclear
Linke et al. (2008)	Low	Low	Low	Low	Unclear	Low	Low
Fabbrini et al. (2010)	Low	Unclear	Low	Low	Unclear	Low	Unclear
Aye et al. (2014)	Low	Low	Low	Low	Unclear	Low	Low
Vittone et al. (2007)	Low	High	Low	Low	Unclear	Low	Low
Vaccari et al. (2007)	Low	Low	High	Low	Unclear	Low	Unclear
Superko et al. (2004)	Low	Low	Unclear	Low	Unclear	Low	Low
Savinova et al. (2015)	Low	Unclear	Low	Low	Unclear	Low	Unclear
Bays et al. (2010)	Low	Low	Low	Low	Unclear	Low	Low
Okabe et al. (2022)	Low	Unclear	Unclear	Low	Unclear	Low	Low
Ko et al. (1998)	Low	High	High	Low	High	Low	High
Kei et al. (2011)	Low	Low	High	Unclear	Unclear	Low	Unclear
Chauhan et al. (2011)	Low	Low	Unclear	Unclear	Unclear	Low	Unclear
Osar et al. (2004)	Low	Low	High	Low	Unclear	Low	Unclear
Westphal et al. (2007)	Low	Low	Unclear	Low	Unclear	Low	Low
Lee et al. (2009)	Low	Low	Low	Low	Unclear	Low	Low

### Data synthesis and statistical analysis

The data were examined using STATA version 12.0 software. Different data types were converted using a predetermined procedure to the mean and standard deviations (S.D.s) (19, 20). For instance, in the absence of standard deviations, we calculated the change using the method below: The definition of standard deviation changes is square root [(S.D. baseline ^2^ + SD final ^2^) - (2R S.D. baseline 2 S.D. final)]. The following formula is used to convert the standard error of the mean (SEM) to standard deviation: S.D. is equal to SEM × √n, where n is the total number of participants in each group. The random-effects model was employed in the meta-analysis of research results. The weighting of the research followed the typical inverse variance technique. The data from the longest time point were used for the analysis, which allowed for the handling of many assessments within a single study group. Using Q Statistics and I-squared (I^2^), the degree of study heterogeneity was evaluated. Insignificant, low, moderate, and high heterogeneity were found with I^2^ values ranging from 0% to 25, 26 to 50%, 5 to 75%, and 76 to 100%, respectively (21). To identify possible causes of heterogeneity, a pre-defined subgroup analysis based on the dosage, duration, and type of the intervention was conducted. A sensitivity analysis was done to determine the contribution of each research to the overall mean difference. In order to establish if there was publication bias, we utilized the official Egger’s test (22).

## Results

**Figure 1:**
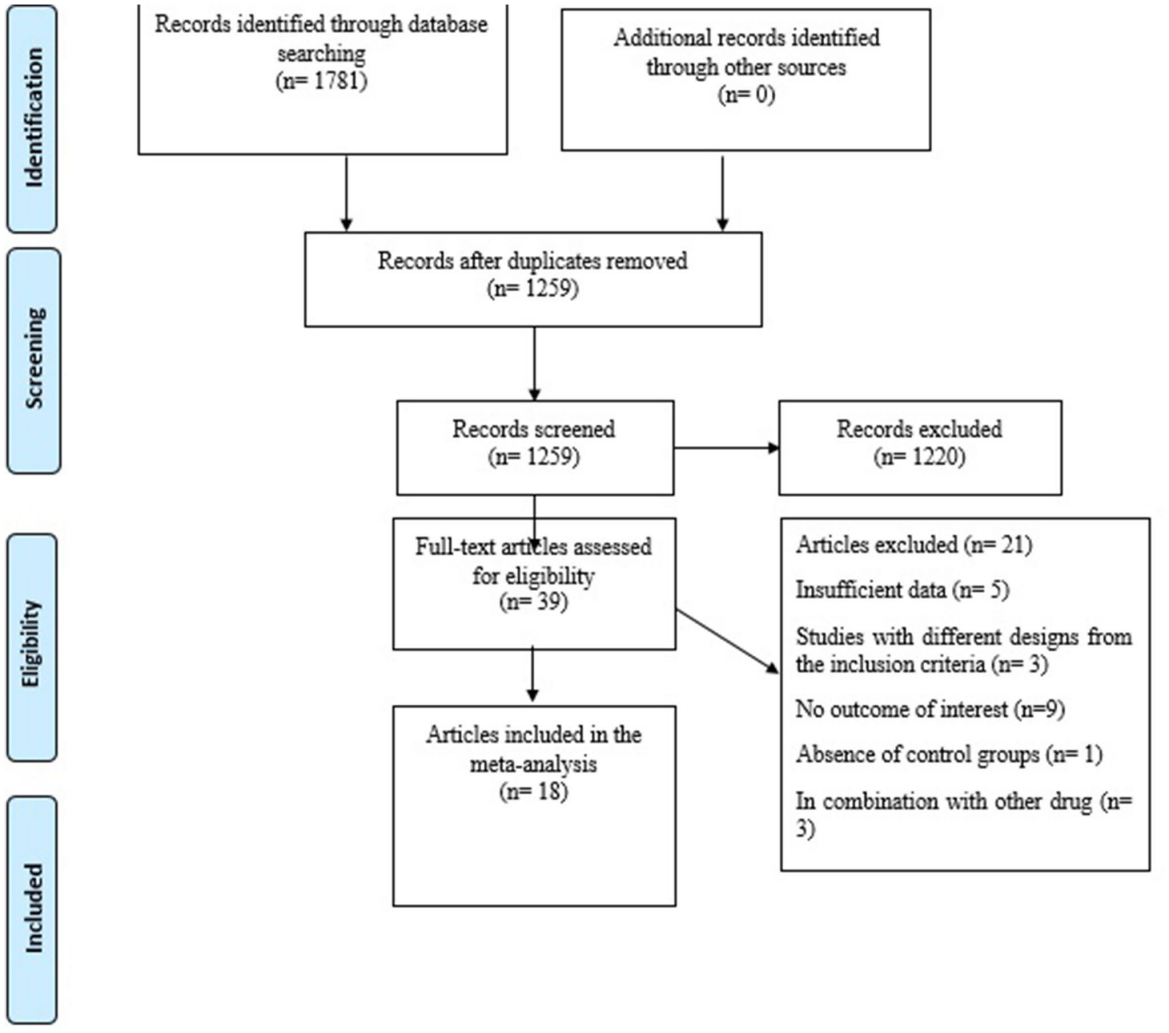
Flow chart of the study, including identification, screening, eligibility, and the final sample included.

Figure 1 depicts a flowchart of the research selection process with exclusion criteria. This value indicates that the aforementioned electronic databases generated 1781 articles. After removing publications with duplicate research, there were 1,259 total. Following an assessment of the research’s titles and abstracts, 1,220 papers were dropped since they did not meet the inclusion requirements. 39 articles were found utilizing the full-text search during the secondary screening. For the reasons listed above, 21 of the investigations were dropped. Finally, 18 papers with 22 treatments arm were included in the quantitative meta-analysis since they matched the qualifying requirements.

### Study characteristics

The features of the pooled articles are shown in Table 2. Our surveys reveal that seven studies have been carried out in USA, 2 articles in the Germany and UK, respectively, and other studies were conducted in Japan, China, India, Egypt, Turkey, Hong Kong, and Greece. All articles were published between 1998 and 2022 and follow up intervention ranged from 4 to 144 weeks. The mean age and percentage of male participants ranged from 31 to 65 years and 0–100%, respectively, at the baseline. The doses prescribed in the studies were between 250 and 3,000 mg per day, and in 4 studies the supplement type was in the form of NE and the rest were in the form of NA. Five studies conducted on patients with metabolic syndrome, five articles on people with a history of cardiovascular disease or dyslipidemia, two studies on each of type 2 diabetes (T2DM) and non-alcoholic fatty liver (NAFLD) as well as healthy samples, and two studies on patients with impaired glucose tolerance and polycystic ovary syndrome.

**Table 2:** Characteristics of eligible studies.

Author (year)	Country	Population	Mean age year	Sex (Male %)	Sample size study (intervention/control)	Follow up of intervention (Weeks)	Type and dose (mg/day) of intervention	Baseline of BMI (kg/m^2^)	Outcomes
El-Kady et al. (2022)	Egypt	NAFLD	45.6	41.9	31/30	12	1,000 mg NE	32.69	BMI, weight, Adiponectin
Liao et al. (2021)	China	Healthy Subjects	37	83.3	12/12	6	300, 600, 1,200 mg NE	22	BMI, weight
Canner et al. (2003)	USA	Metabolic Syndrome and Healed Myocardial Infarction	NR	NR	964/2468	48	2000 mg NA	NR	BMI
Linke et al. (2008)	Germany	Patients with impaired glucose tolerance	45.5	70	30/30	24	1,000 mg NA	37.9	BMI, weight, Adiponectin, Leptin
Fabbrini et al. (2010)	USA	NAFLD	43	30	9/9	16	2000 mg NA	35.8	BMI, weight
Aye et al. (2014)	UK	Polycystic ovary syndrome	31	0	13/12	12	1,000 mg NA	35.8	BMI
Vittone et al. (2007)	USA	Patients with metabolic syndrome	54	86.2	80/80	144	2000 mg NA	29.7	BMI
Vaccari et al. (2007)	USA	Patients with metabolic syndrome	32	56	30/15	52	1,000 mg NA	29.7	BMI, Adiponectin
Superko et al. (2004)	USA	Hypercholesterolemic subjects	53	71.6	60, 59/61	14	1,500, 3,000 mg NA	28	BMI
Savinova et al. (2015)	USA	Patients with metabolic syndrome	47	57	14/14	16	2000 mg NA	32.7	BMI
Bays et al. (2010)	USA	Dyslipidemic patients without metabolic syndrome	57.7	62.4	221, 320/110, 160	24	2000 mg NA	31.5	BMI
Okabe et al. (2022)	Japan	Healthy Subjects	42.9	26.6	15/15	16	250 mg NE	21.3	BMI, weight
Ko et al. (1998)	Hong Kong	T2DM	59.2	36.3	32/30	12	750 mg NA	25.7	BMI
Kei et al. (2011)	Greece	Dyslipidemic patients	58	46.6	30/30	12	2000 mg NA	29.1	weight
Chauhan et al. (2011)	India	Hyperlipidemic patients	NR	NR	17/20	12	2,250 mg NA	NR	weight
Osar et al. (2004)	Turkey	Patients with Poorly Controlled Type 2 Diabetes Mellitus	58	46.6	15/15	4	3,000 mg NE	30	weight
Westphal et al. (2007)	Germany	Patients with metabolic syndrome	55	100	20/10	6	1,500 mg NA	32.4	Adiponectin, Leptin
Lee et al. (2009)	UK	Patients with coronary artery disease	65	94	22/29	48	2000 mg NA	31	Adiponectin

BMI, body mass index; NAFLD, Nonalcoholic fatty liver disease; TD2M, Type 2 diabetes; NR, not report; NA, Nicotinic acid; NE, Nicotinamide.

The findings of the evaluation of the eligible studies’ quality are shown in Table 1. Additionally, a score of 8.8 (very good quality) was determined after the GRADE score system was used to assess the quality of the current meta-analysis.

### Meta-analysis results

**Figure 2:**
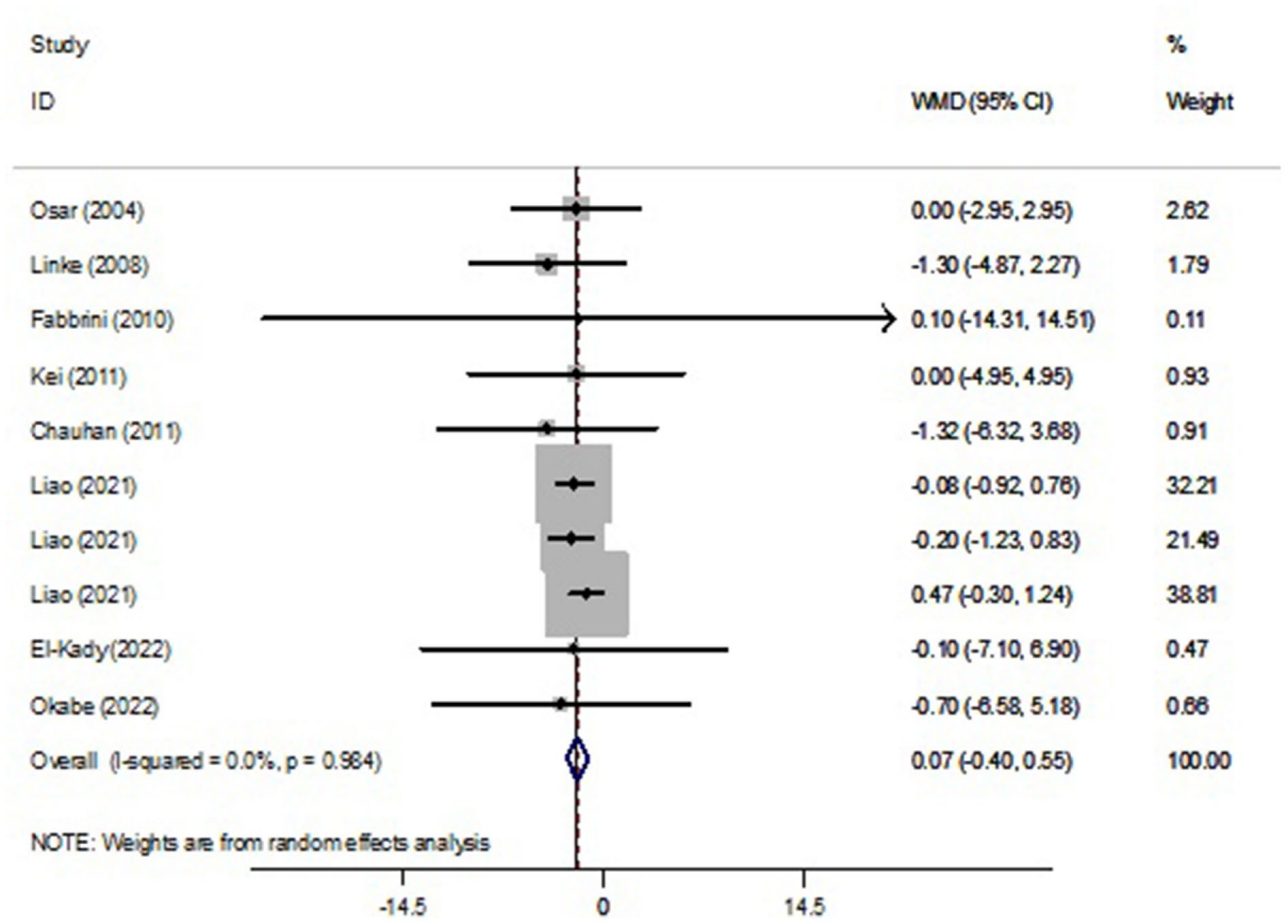
Forest plot of randomized controlled trails investigating the effects of NAD+ precursor supplementation on weight (kg).

**Figure 3:**
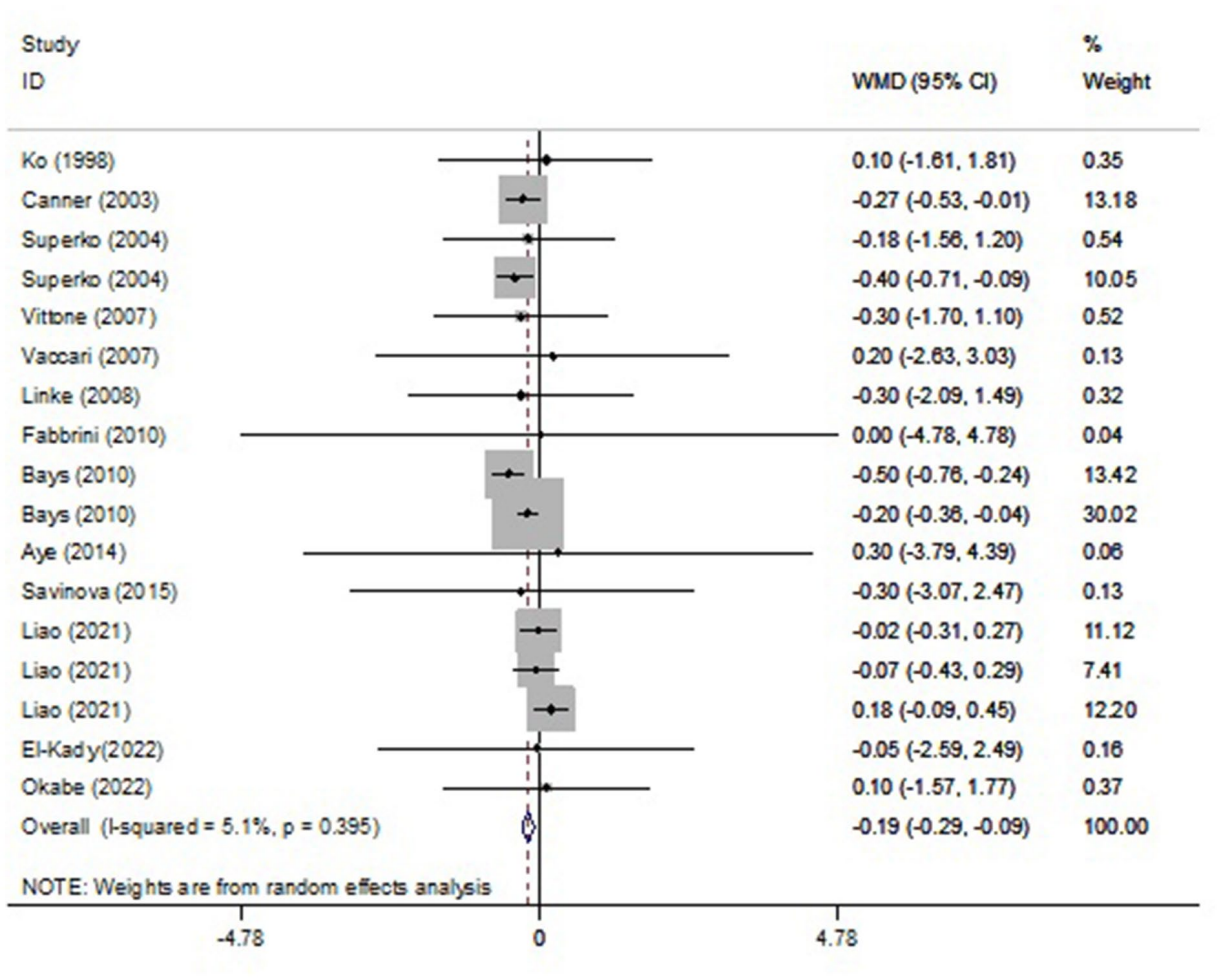
Forest plot of randomized controlled trails investigating the effects of NAD+ precursor supplementation on body mass index (BMI) (kg/m^2^).

**Figure 4:**
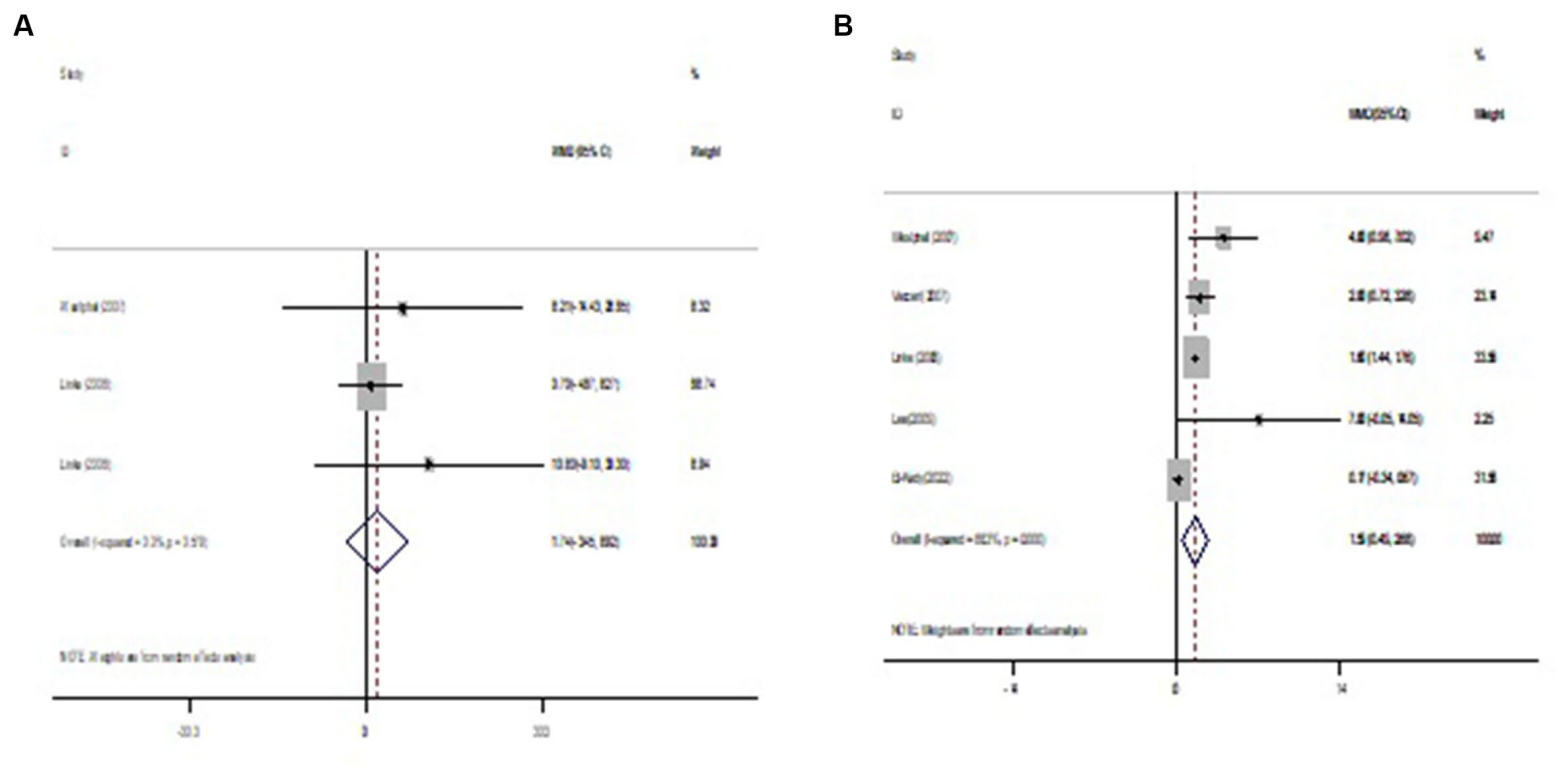
Forest plot of randomized controlled trails investigating the effects of NAD+ precursor supplementation on **(A)** leptin (ng/ml) and **(B)** adiponectin (μg/mL).

Pooled findings from the random-effects model indicated that body mass index (BMI) (weighted mean difference (WMD): −0.19 kg/m^2^, 95% confidence interval (CI): −0.29 to −0.09, *p* < 0.001) were significantly reduced after NAD+ precursor supplementation compared to control group. However, no significant effect was observed on body weight (WMD: 0.07 kg, 95% CI: −0.40 to 0.55, *p* = 0.766), leptin (WMD: 1.74 ng/mL, 95% CI: −3.45 to 6.92, *p* = 0.512) compared to the control group. Also, Pooled findings indicated that compared to the control group, adiponectin (WMD: 1.59 μg/mL, 95% CI: 0.49 to 2.68, *p* = 0.004) was significantly increased after with NAD+ precursor supplementation. Furthermore, significant heterogeneity was found among the studies for adiponectin (Cochran *Q* test, *p* < 0.001, I^2^ = 88.3%). However, low heterogeneity was reported for weight (Cochran Q test, *p* = 0.984, I^2^ = 0.0%), BMI (Cochran Q test, *p* = 0.395, I^2^ = 5.1%), and leptin (Cochran Q test, *p* = 0.579, I^2^ = 0.0%) (Figures 2–4).

### Subgroups analysis

The findings of the subgroup also show the greater effect of NAD+ precursor supplementation on BMI decrease in a dose ≥2 g and a duration of intervention >12 weeks. In addition, subgroup analysis showed that BMI reduction was greater when receiving nicotinic acid (NA) supplementation than nicotinamide (NE) supplementation (Supplementary Table).

### Meta-regression

Meta-regression between NAD+ precursor and absolute mean differences in body weight and BMI based on dosage and duration of intervention was performed. Only, there was a significant relationship between dose of intervention with changes in BMI (coefficient (Coef) = −0. 0001847, *p* = 0.033). However, meta-regression analysis did not show a significant linear relationship between dose and duration of intervention with weight changes (Supplementary Figures S1, S2).

### Sensitivity analysis

To discover the effect of each article on the pooled effect size for the levels of weight, BMI, adiponectin, and leptin, we step-by-step discarded each trial from the analysis. The leave-one-out sensitivity analysis indicated the robustness of the results (Supplementary Figure S3).

### Publication bias

Evaluating the publication bias by visual inspection of the funnel, no evidence for publication bias based on the Egger’s tests was detected for weight (*p* = 0.531), BMI (*p* = 0.621), adiponectin (*p* = 1.00), and leptin (*p* = 0.602; Supplementary Figure S4).

## Discussion

The results of our systematic review and meta-analysis showed that nicotinamide adenine dinucleotide (NAD+) precursor supplementation has an effect on lowering BMI and increasing adiponectin in humans compared with control groups. Furthermore, there was a significant relationship between doses of intervention with changes in BMI. The findings of the subgroup also show the greater effect of NAD+ precursor supplementation on BMI decrease in a dose ≥2 g and a duration of intervention >12 weeks.

Currently, there is no meta-analysis on the effect of NAD+ precursors on obesity in the human body. Earlier meta-analysis investigated and described the effect of NAD+ precursor supplementation on improving TG, TC, LDL, and HDL levels in humans, but resulted in hyperglycemia, compared with placebo or no treatment (23, 24). Animal studies evaluating obese mice have shown an association between NAD+ supplementation and improved indices of obesity as well as molecular regulation of adipocytes (25, 26).

Nicotinamide adenine dinucleotide (NAD+) is an important molecule in energy and signal transduction, besides acting as substrate for enzymes such as sirtuins, poly-ADP ribose polymerases (PARPs), and cyclic ADP ribose synthetases that regulate key cellular processes of energy metabolism, DNA damage repair, and calcium signaling (27). Improving NAD+ availability *via* NAD+ precursor supplementation has emerged as a potential strategy to augment tissue-specific NAD+ homeostasis and improve physiological function (28). Thus, NAD+ supplementation therapy is a new treatment for obesity in recent years because acts *via* activation of the NAD + -dependent sirtuin enzyme family, thereby regulating oxidative metabolism (29, 30).

NAD+ precursors such as nicotinamide riboside (NR) and nicotinamide mononucleotide (NMN) is a recently discovered vitamin B3, are available as over-the-counter dietary supplements, and oral supplementation with these precursors reduction in fat mass and an increase in lean mass; improved glucose tolerance and alleviated adipose tissue inflammation in pre-clinical models with obesity (26). Remie et al. (31) has demonstrated that NAD+ supplementation therapy with 1,000 mg/d for 6 weeks in healthy overweight or obese men and women increased skeletal muscle NAD+ metabolites, affected skeletal muscle acetylcarnitine metabolism, and induced minor changes in body composition.

Consistent with the effect of NAD+ precursor supplementation on body composition observed by Remie et al. (31), we have shown lowering BMI at 0.19 kg/m^2^ and BMI decrease was higher in a dose ≥2 g and a duration of intervention >12 weeks. In another study in 2018, which was conducted to investigate dietary nicotinamide riboside (NR) supplementation in a 12-week period on the improvement of insulin sensitivity and other metabolic parameters in obese and insulin-resistant men, the results showed that NR in doses of 2 g/day had no effect on resting energy expenditure, lipolysis, lipid oxidation, or body composition (32). In a study, with the aim of determining the effect of long-term NR supplementation on increasing mitochondrial biogenesis and metabolic health on twenty monozygotic twins discordant with BMI with an increasing dose of NR (250 to 1,000 mg per day) for 5 months, NR did not improve obesity or metabolic health (33). Udin et al. (34) evaluated the effects of NAD+ supplementation in rats fed a high-fat diet. The results showed that NAD+ supplementation reduced the BMI of the rats, suggesting that NAD+ supplementation may be an effective strategy for weight loss and improving metabolic health. In another animal study on diet-induced obese rats, NA decreased visceral adipose tissue and improved adiponectin levels and lipid profile (35). However, a study on developing rats did not show any useful results regarding the effect of NE on factors related to obesity (36).

However, despite the promising result for BMI lowering in the face of supplementation, no significant effect was observed on body weight. Despite previous evidence from preclinical studies on promoting resistance to weight gain no significant effect on body weight in human studies was observed in our meta-analysis. We believe that the discrepancy between the results observed for the BMI and body weight outcomes is related to the smaller number and consequent smaller effect size of studies selected for the body weight outcome in relation to the BMI outcome (37). Moreover, we observed that for the outcome of body weight, the majority of weight of the studies obtained from the effect size was concentrated in the evidence demonstrated by Liao et al. (38), which was responsible for 92.5% of the overall effect (% weight), this can overestimate the total effect.

To investigate the role of the effect of NAD+ precursor supplementation on obesity, we evaluated leptin and adiponectin. Adipose tissue is a metabolic and endocrine organ that secretes a number of adipokines that contribute to the etiology of obesity-related metabolic complications (39, 40). Leptin is a hormone produced by adipose tissue that plays an important role in regulating appetite and energy metabolism. Elevated leptin levels are associated with suppressed appetite and increased energy expenditure, while reduced leptin levels are associated with increased appetite and decreased energy expenditure (41). Adiponectin, on the other hand, is a hormone secreted by fat cells that has been linked to the regulation of metabolism and insulin sensitivity (42). Dysfunction of leptin signaling and reduced adiponectin levels may contribute to the development of obesity. In our review we observed no effect on leptin levels. In contrast, increased levels were observed in patients who used supplementation. Elevated levels of adiponectins in humans may correlate with inflammation or the body’s reaction to exacerbation on contact with new substances, in the case of NAD+ supplementation, so that in the supplemented group there was an increase in its levels, as an attempt to combat the effects of pro-inflammatory cytokines, there is an increase in adiponectins (43). In addition, the fact that a low BMI is associated with an increase in adiponectin can be explained, identifying that the secretion of this adipokine is related to the quality, not the quantity, of adipose tissue, and in addition to BMI, in situations of greater age this also tends to occur (42).

Another interesting finding regarding the effects of NAD+ precursors on BMI was their greater magnitude with the use of NA when compared to NE. However, it is important to highlight that only five RCTs evaluating the effect of NE supplementation on BMI were included in this meta-analysis. In fact, when compared to NA, NE has been less studied regarding its effect on BMI. Thus, more studies evaluating the effect of NE supplementation would be important to better investigate its therapeutic potential in obesity outcomes, allowing a better comparison in relation to other NAD+ precursors. In addition, unlike NE, NA reduces the levels of lipid profiles, including cholesterol and triglycerides, which can explain its greater effect on BMI and be effective on factors related to obesity.

Our study had some limitations that jeopardized the extraction of robust conclusions. Clinically and statistically significant heterogeneities was found for adiponectin. These may be explained by the differences in the intervention-specific factors (e.g., type, dose, administration route, and duration of drugs) and weight-specific factors (e.g., age, sex, physiology, genetics, familial history, race/ethnicity, physical activity, socioeconomic status, dietary intakes, and drug, tobacco, or alcohol consumption) (44). Nonetheless, we attempted to identify some possible sources of heterogeneity in data by performing a subgroup analysis. In addition, lack of registration of the current study in PROSPERO due to time limit was another limitation of this study.

Despite its limitations, the current study has several positive features: a rigorous methodology was used based on the PRISMA guidelines; a comprehensive literature search included different independent databases; search, selection, and data extraction applied to the selected studies were performed separately, and in duplicate, by two researchers; a third party was accessed to solve disagreements. Furthermore, the present study likely included the largest effect size for each outcome assessed at obesity.

## Conclusion

The finding of this paper showed that NAD+ precursor supplementation has an effect on lowering BMI and increasing adiponectin in humans compared with control groups. However, no significant effect was observed on body weight and leptin. Given the evidence of decreased BMI in humans and increased adiponectin, it is important to emphasize that NAD+ supplementation should not be seen as a one-time solution for weight loss and should be combined with healthy eating habits and exercise under professional guidance from a dietitian.

## Data availability statement

The raw data supporting the conclusions of this article will be made available by the authors, without undue reservation.

## Ethics statement

This study was approved by the research council and ethics committee Shahid Beheshti University of Medical Sciences, Tehran, Iran.

## Author contributions

MS and YB contributed in conception, design, and statistical analysis. MS, YB, ST, MG, and NS contributed in data collection and manuscript drafting. MS supervised the study. All authors contributed to the article and approved the submitted version.
