# Supplementary material for: The effects of NAD+ precursor (nicotinic acid and nicotinamide) supplementation on weight loss and related hormones: a systematic review and meta-regression analysis of randomized controlled trials
Source: Front Nutr. 2023 Oct 3;10:1208734. doi: 10.3389/fnut.2023.1208734 (PMC10579603; doi:10.3389/fnut.2023.1208734)
Supplement: Supplementary file 1 [file Table_1.docx]

**Supplemental Table 1-** Meta-analyses showing the effect of NAD+ precursor supplementation on several subgroups (all analyses were conducted using random effects model).

|  | | | | **Heterogeneity** |  |  |
| --- | --- | --- | --- | --- | --- | --- |
|  | | **No. of**  **Treatment arms** | **WMD^1^ (95%CI)** | | ***I^2^* (%)** | ***P* within group** |
| **Weight (kg)** | |  |  | |  |  |
| ***Duration*** | *≤ 12 weeks* | 7 | **0.10 -0.38 0.59** | | ***0.0*** | ***0.943*** |
|  | *> 12 weeks* | 3 | **-1.09 -4.07 1.90** | | ***0.0*** | ***0.972*** |
| ***Dosage*** | *≥ 2 g* | 4 | **-3.26 -2.49 1.97** | | ***0.0*** | ***0.975*** |
|  | *< 2 g* | 6 | **0.09 -0.40 0.58** | | ***0.0*** | ***0.840*** |
| **Type of intervention** | *Nicotinic acid (NA)* | 4 | **-0.94 -3.41 1.53** | | ***0.0*** | ***0.974*** |
|  | *Nicotinamide (NE)* | 6 | **0.11 -0.37 0.60** | | ***0.0*** | ***0.915*** |
| **BMI ( kg/m^2^)** | |  |  | |  |  |
| ***Duration*** | *≤ 12 weeks* | 6 | **0.05 -0.12 0.22** | | ***0.0*** | ***0.908*** |
|  | *> 12 weeks* | 11 | **-0.29 -0.40 -0.18** | | ***0.0*** | ***0.914*** |
| ***dosage*** | *≥ 2 g* | 7 | **-0.30 -0.41 -0.18** | | ***0.0*** | ***0.639*** |
|  | *< 2 g* | 10 | **0.04 -0.13 0.21** | | ***0.0*** | ***0.994*** |
| **Type of intervention** | *Nicotinic acid (NA)* | 12 | **-0.29 -0.40 -0.18** | | ***0.0*** | ***0.944*** |
|  | *Nicotinamide (NE)* | 5 | **0.05 -0.12 0.22** | | ***0.0*** | ***0.822*** |
| **Adiponectin (µg/ml)** | |  |  | |  |  |
| **Type of intervention** | *Nicotinic acid (NA)* | 4 | **2.01 1.07 2.94** | | ***40.3*** | ***0.170*** |
|  | *Nicotinamide (NE)* | 1 | **0.17 -0.34 0.67** | | ***-*** | ***-*** |
