# Supplementary material for: The effects of NAD+ precursor (nicotinic acid and nicotinamide) supplementation on weight loss and related hormones: a systematic review and meta-regression analysis of randomized controlled trials
Source: Front Nutr. 2023 Oct 3;10:1208734. doi: 10.3389/fnut.2023.1208734 (PMC10579603; doi:10.3389/fnut.2023.1208734)
Supplement: Supplementary file 2 [file Data_Sheet_1.docx]

|  |  |
| --- | --- |
| **Supplementary Figures 1**. Meta-regression analysis encompassing weight changes according to the duration of intervention (weeks) and dose of intervention (g). | |

|  |  |
| --- | --- |
| **Supplementary Figures 2**. Meta-regression analysis encompassing BMI changes according to the duration of intervention (weeks) and dose of intervention (g). | |

| A)   | B)   |
| --- | --- |
| C)   | D)   |

**Supplementary Figures 3**. Sensitivity analysis of the weighted mean difference (WMD) for A) weight, B) BMI, C) leptin, and D) adiponectin changes. *BMI*: Body mass index, *WC*: Waist circumference

| A)   | B)   |
| --- | --- |
| C)   | D)   |

**Supplementary Figures 4**. Funnel plots for evaluation of publication bias of A) weight, B) BMI, C) leptin, and D) adiponectin changes. *BMI*: Body mass index, *WC*: Waist circumference
