# Supplementary material for: The effects of NAD+ precursor (nicotinic acid and nicotinamide) supplementation on weight loss and related hormones: a systematic review and meta-regression analysis of randomized controlled trials
Source: Front Nutr. 2023 Oct 3;10:1208734. doi: 10.3389/fnut.2023.1208734 (PMC10579603; doi:10.3389/fnut.2023.1208734)
Supplement: Supplementary file 3 [file Data_Sheet_2.docx]

***Supplementary Appendix.***

***S1. Search strategy***

***PubMed/Medline***

(“NAD"[MeSH Terms] OR "NAD precursor"[Title/Abstract] OR "Nicotinic Acids"[MeSH Terms] OR "Nicotinic Acid"[Title/Abstract] OR "NA"[Title/Abstract] OR "Niacin"[MeSH Terms] OR "Niacin"[Title/Abstract] OR "Niacinamide"[MeSH Terms] OR "Niacinamide"[Title/Abstract] OR "Nicotinamide"[Title/Abstract] OR "NAM"[Title/Abstract] OR "Nicotinamide Riboside"[Title/Abstract] OR "NR"[Title/Abstract] OR "Nicotinamide Mononucleotide"[MeSH Terms] OR "Nicotinamide Mononucleotide"[Title/Abstract] OR "NMN"[Title/Abstract]) AND (“Adipokines”[MeSH] OR leptin [Mesh] OR Adiponectin [Mesh] OR “Body Mass Index”[ All Fields] OR “Body Mass Index”[Mesh] OR BMI [All Fields] OR weight [All Fields] OR Body Weight [Mesh]) AND ("Clinical Trials as Topic"[Mesh] OR "Cross-Over Studies"[Mesh] OR "Double-Blind Method"[Mesh] OR "Single-Blind Method"[Mesh] OR "Random Allocation"[Mesh] OR RCT[All Fields] OR "Clinical Trial" [Publication Type] OR "Controlled Clinical Trials as Topic"[Mesh] OR "Intervention Studies"[ All Fields] OR intervent*[All Fields] OR Trial[All Fields] OR "controlled trial"[ All Fields] OR randomize*[All Fields] OR randomise*[All Fields] OR random*[All Fields] OR placebo[All Fields] OR assignment[All Fields])
